# Maxillomandibular impalement due to a gardening hoe strike to the face

**DOI:** 10.4103/0974-2700.66561

**Published:** 2010

**Authors:** Elio Hitoshi Shinohara, Shajadi Carlos Pardo-Kaba, Marcelo Zillo Martini, Carlos Henrique Hueb

**Affiliations:** Hospital Israelita Albert Einstein and Post-Graduate Program Oral and Maxillofacial Surgery Branch, Araçatuba Dental School-UNESP and UNINOVE Dental School, São Paulo, Brazil; 1 Department of Oral and Maxillofacial Surgery, Hospital Estadual “José Pangella”-Vila Penteado-SUS/SP. Former: Conjunto Hospitalar do Mandaqui SUS/SP. Sao Paulo, Brazil; 2 Dental School Sao Francisco University, Bragança Paulista and Oral Diagnosis Program. FOUSP, Sao Paulo, Brazil; 3 Oral and Maxillofacial Surgery Service, Ermelino Matarazzo Hospital. SMS/SP. Sao Paulo, Brazil

**Figure 1:**
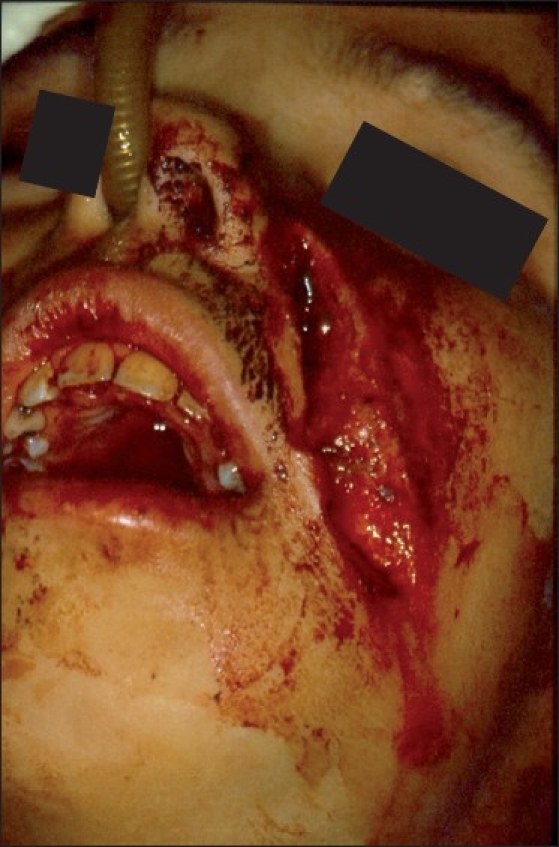
Open wound in the left zygomatic region extending in the posteroanterior direction and reaching the left mandibular base

**Figure 2:**
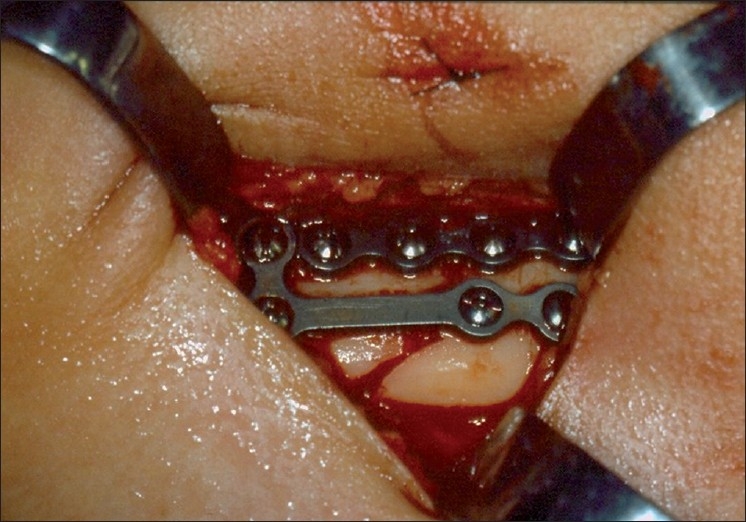
Mandibular fracture stability was achieved with two titanium miniplates and 2.0-mm screws

**Figure 3:**
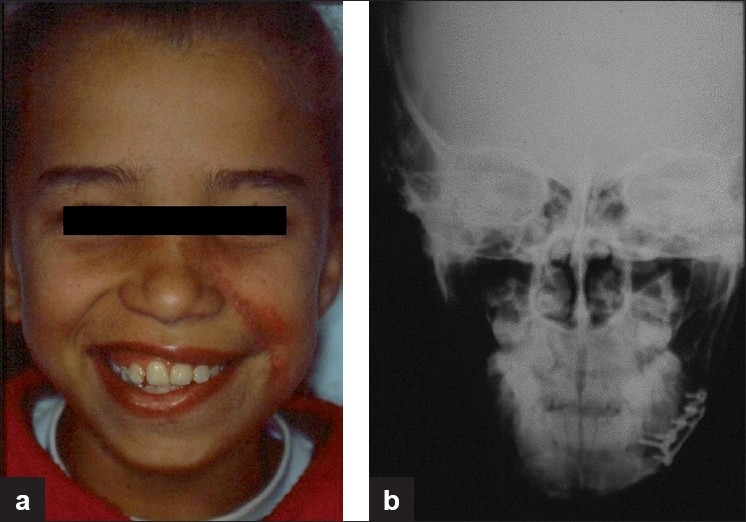
Clinical (a) and radiographic (b) aspects after 7 months

Penetrating wounds of the face occur less frequently than in other regions of the body due to protective reflexes.[1] Facial impalement reports are mainly described due to the bizarre and atypical nature of the wounds because treatment protocols and surgical maneuvers employed are the same as usual. According to Eppley (2002),[2] these impalement injuries describe unusual objects and circumstances in which a body part is either partially embedded (one end sticking out) or transected (through-and-through) by a foreign material. These instruments or materials in their typical application have remote possibilities of causing such types of injuries. Wounds involving these kinds of instruments occur most commonly in the trunk and members, due to the larger exposed area, and are rarely seen in facial injuries. Facial impalement injuries caused by knives,[1] fan blade,[3] and even a model aircraft[4] are already described in the literature, but facial impalement injuries caused by being hit with a gardening hoe have not been described. Our patient was a 9-year-old white girl who presented to our Emergency Department with a severe facial wound caused by an accidental strike with a gardening hoe handled by her grandmother while trying to protect her from a dog attack. The vital signs, airway, breathing, and circulation parameters were all within normal limits. The facial trauma was focused on the lower third of the face and a detailed examination revealed a cut, bruised wound in the left-sided zygomatic body, extending in the posteroanterior direction and reaching the left mandibular base [Figure 1], with an open fracture of the left mandibular body and a fracture fissure in the left side of the posterior maxilla. Radiographic examination did not contribute to surgical planning as the child was constantly moving her head during radiographic examination. Because of the superficial nature of the wounds and the good visual access to the open fracture, we chose not to repeat the radiograph exam. The patient underwent emergency surgery for reduction and fixation of the mandibular fracture. The mandibular fracture was reduced and stabilized with two titanium 2.0-mm miniplates and monocortical screws (Medicon^©^ Tuttlingen-Germany) [Figure 2]. The patient was put on antibiotics and her tetanus vaccine status was updated. The patient was discharged after 72 h, and 7 months later her dental occlusion remained normal, and the only sequel she presented with was a deficiency in facial motricity caused by cicatricial retraction and traumatic lesion of the facial nerve [Figure 3a and b].
